# The complete mitochondrial genome of *Symplocos tanakana*

**DOI:** 10.1080/23802359.2025.2528579

**Published:** 2025-07-10

**Authors:** Jianfeng Cao, Bingqin Wang, Yudong Gan, Chao Wu, Boxing Cheng

**Affiliations:** a School of Biological Sciences, Guizhou Education University, Guiyang, P.R. China; b Guizhou Key Laboratory of Bioresources Development and Utilization, Guizhou Education University, Guiyang, P.R. China

**Keywords:** *Symplocos tanakana*, mitochondrial genome, phylogenetic analysis

## Abstract

*Symplocos tanakana* Nakai (1918) is a deciduous shrub or small tree with multiple applications. It is native to China and belongs to the Symplocaceae family. This study presents the complete mitochondrial genomic sequence of *S. tanakana.* This is the first member of the Symplocaceae family for which the complete mitochondrial genomic sequence is being reported. The mitochondrial genome was assembled into a single 877,273-bp circular molecule that contains 290 open reading frames (ORFs) and 68 annotated genes, including 38 protein-coding, 3 ribosomal RNA, and 27 transfer RNA genes. Phylogenetic analysis of the mitochondrial genomes of 33 species from 17 plant families using the maximum likelihood method showed evolutionary relationships. These findings enhance our understanding of the *S. tanakana* mitochondrial genomic features and provide a basis for the development of *S. tanakana* molecular markers.

## Introduction

*Symplocos tanakana* Nakai (1918) is a deciduous shrub or small tree that is native to China and belongs to the genus *Symplocos* of the Symplocaceae family. This plant has a wide range of applications. Due to its resilience under harsh conditions, *S. tanakana* thrives in salty, barren, and extremely dry soils, as well as rocky environments (Gaur 1999). Additionally, *S. tanakana* is widely recognized as a valuable source of biodiesel and edible oil, attributed to its high fruit yield and substantial 30–40% seed oil content (China Oil Plant Editorial Committee 1987; Liu et al. 2010). This important plant contains various bioactive compounds, including flavonoids, triterpenoids, saponins, iridoids, and tannins, which confer multiple medicinal applications (Seong et al. 2023). Its root and bark are used for treating gastrointestinal ailments (Chopra et al. 1992), bleeding gums, ulcers (Semwal et al. 2011), and diabetes (Na et al. 2006). Furthermore, *S. tanakana* is used as an ornamental plant owing to its dark green deciduous leaves, white fragrant hermaphrodite flowers, and attractive blueberry-like fruits (Manandhar 2002; Liu et al. 2012).

Although the chloroplast genome of S. tanakana has been sequenced, no studies have reported the mitochondrial genome of any Symplocaceae species. In this study, we determined the complete mitochondrial genome of *S. tanakana* for the first time using the Illumina NovaSeq 6000 and Nanopore platforms. Then, we performed a phylogenetic analysis and compared the complete mitochondrial genomes of *S. tanakana* and related genera, the results would aid in the comprehension of the mitochondrial genomic features of *S. tanakana*.

## Materials and methods

### DNA sequencing and genome assembly

**Figure 1.**
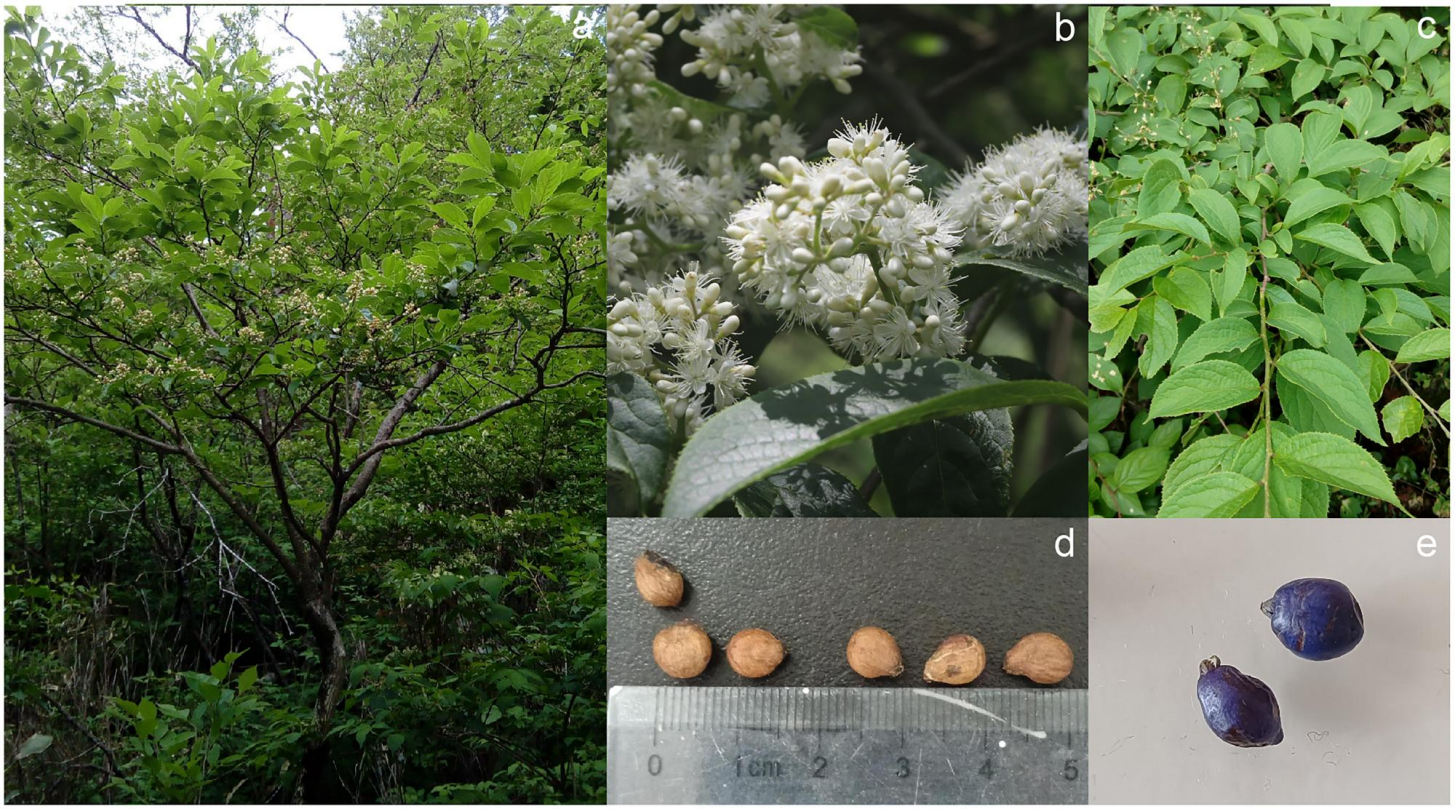
Morphological characteristics of *symplocos tanakana* (a) whole plant, (b) inflorescence, (c) leaves, (d) seeds, and (e) fruits. This photograph was captured by the first author (jianfeng cao).

Whole *S. tanakana* plants were collected from the Nantun village Farm in Weining County, Guizhou Province, China (26° 48′ N, 104° 19′ E). It is a deciduous shrub or small tree (Figure 1) with ovate, elliptic-obovate, or broadly obovate leaf blades and ovoid-globose bluish drupes. Flowering occurs from April to June, and the fruiting stage occurs from September to October. *S. tanakana* is neither endangered nor protected. Ethical approval for this study was obtained from the Institutional Bioethics Committee of Guizhou Education University.

Young *S. tanakana* leaves were collected, cleaned, immediately frozen in liquid nitrogen, and stored at −80 °C. The voucher specimens of *S. tanakana* were deposited at the herbarium of Guizhou Key Laboratory of BioResources Development and Utilization (https://www.gznc.edu.cn/, Shichao Lan and 397530497@qq.com) under the voucher number ST-GZ14223002. Mitochondria were isolated from the leaves using density-gradient centrifugation and digested with DNase I (Promega, Madison, WI, USA) to eliminate genomic DNA contamination (Li et al. 2000). The quality of *S. tanakana* mitochondrial DNA was determined using the Qubit fluorometer (Thermo Fisher, Massachusetts, USA) and agarose gel electrophoresis.

A full-length mitogenome sequence was obtained in this study by using both short-read (Illumina) and long-read sequencing (PacBio Sequel II) technologies. First, approximately 2 µg of mitochondrial DNA was converted into SMRTbell libraries using the Express Template Prep Kit 2.0 from PacBio according to the manufacturer’s protocol (Dierckxsens et al. 2017). The samples were pooled into a single multiplexed library, and the size was selected using Sage Sciences BluePippin System with a size selection cutoff of 5000 (BP start value). The size-selected SMRTbell library was annealed and bound according to the SMRT link setup and sequenced on Sequel II. Approximately 1 µg of mitochondrial DNA was sonicated using the Covaris M220 system to shear it into approximately 500 bp fragments. The sonicated DNA was purified using a TIANgelMidi Purification Kit, and a sequencing library was constructed using the NEBNext^®^Ultra^™^ DNA Library Prep Kit for Illumina^®^ (New England Biolabs, Ipswich, MA, England) according to the manufacturer’s instructions. The libraries were sequenced using an Illumina NovaSeq 6000 system with 150 bp paired-end reads lengths. Short raw reads were checked using Fast QC and trimmed using Trimmomatic (TruSeq-PE. fa:2:30:10 LEADING:3 TRAILING:3 MINLEN:75). The long raw reads were base-called using Albacore v2.1.7 (mean_qscore > 7) with barcode demultiplexing and converted to FASTA format using Samtools Fasta (http://www.htslib.org/doc/samtools.html).

The mitogenomes of higher plants showed pronounced variation in both structure and sequence. We improved the reliability of the assembly by using two strategies to assemble the *S. tanakana* mitogenome. First, the short, clean reads were de novo assembled using GetOrganelle v1.6.4, and potential mitochondrial contigs were extracted through alignment against the mitochondrial protein-coding genes from the plant mitogenome database (ftp://ftp.ncbi.nlm.nih.gov/refseq/release/mitochondrion/) with BLASTv2.8.1+. Putative long mitochondrial reads were baited by mapping the PacBio long reads to potential mitochondrial contigs using BLASR v5.1 (Koren et al. 2017) and assembled using Canu v2.1.1. Next, all PacBio long reads were directly assembled de novo using Canu v2.1. Subsequently, BWA was used to map short, clean reads to draft contigs, and the draft contigs were improved using Pilon v1.22. Then, MUMmer 3.23 was used to check whether these contigs were circular, and the corrected contigs obtained from the two assembly strategies were aligned, which provided identical results. Thus, we successfully obtained a master circle of the *S. tanakana* mitogenome.

### Gene annotation

The mitochondrial genes were annotated using the online PMGA (http://www.1kmpg.cn/pmga) (Li et al. 2025) and the GeSeq tool (Tillich et al. 2017), with the default stop parameter to predict protein-coding, transfer RNA (tRNA), and ribosome RNA (rRNA) genes. The position of each coding gene was determined by performing BLAST searches against reference mitochondrial genes. Manual corrections of genes for start/stop codons and intron/exon boundaries were performed using the Snap Gene Viewer by referencing the reference genome (Greiner et al. 2019). A mitochondrial genome map of *S. tanakana* was drawn using OGDRAW. Functional annotations were performed using sequence similarity BLAST searches with a typical E-value cutoff of 10^−5^ against the publicly available protein databases, namely (Altschul et al. 1990), the NCBI non-redundant (Nr) protein database, Swiss-Prot (Magrane and UniProt Consortium 2011), Clusters of Orthologous Groups (COGs) (Tatusov et al. 1997), Kyoto Encyclopedia of Genes and Genomes (KEGG) (Kanehisa et al. 2004), and Gene Ontology (GO) terms (Ashburner et al. 2000).

### Phylogenetic analysis

The phylogenetic relationships of *S. tanakana* were evaluated by aligning its complete mitochondrial genome sequences with those of 32 other species from 24 genera across 16 families using MAFFT (Katoh and Standley 2013). To establish the data matrix, a maximum likelihood tree was constructed based on the following 13 conserved protein-coding genes of *S. tanakana: atp1*, *atp4*, *atp9*, *nad2*, *nad3*, *nad4*, *nad4L*, *ccmB*, *ccmC*, *ccmFC*, *ccmFN*, *cob*, and *matR*. This was performed using PhyML v3.0 (Guindon et al. 2010). The model GTR+I + G was selected for ML analyses with 1000 bootstrap replicates to calculate the bootstrap values (BS) of the topology. The results tree were treated with iTOL 3.4.3 (Letunic and Bork 2016).

## Results

### Genome sequence assembly and characterization

**Figure 2.**
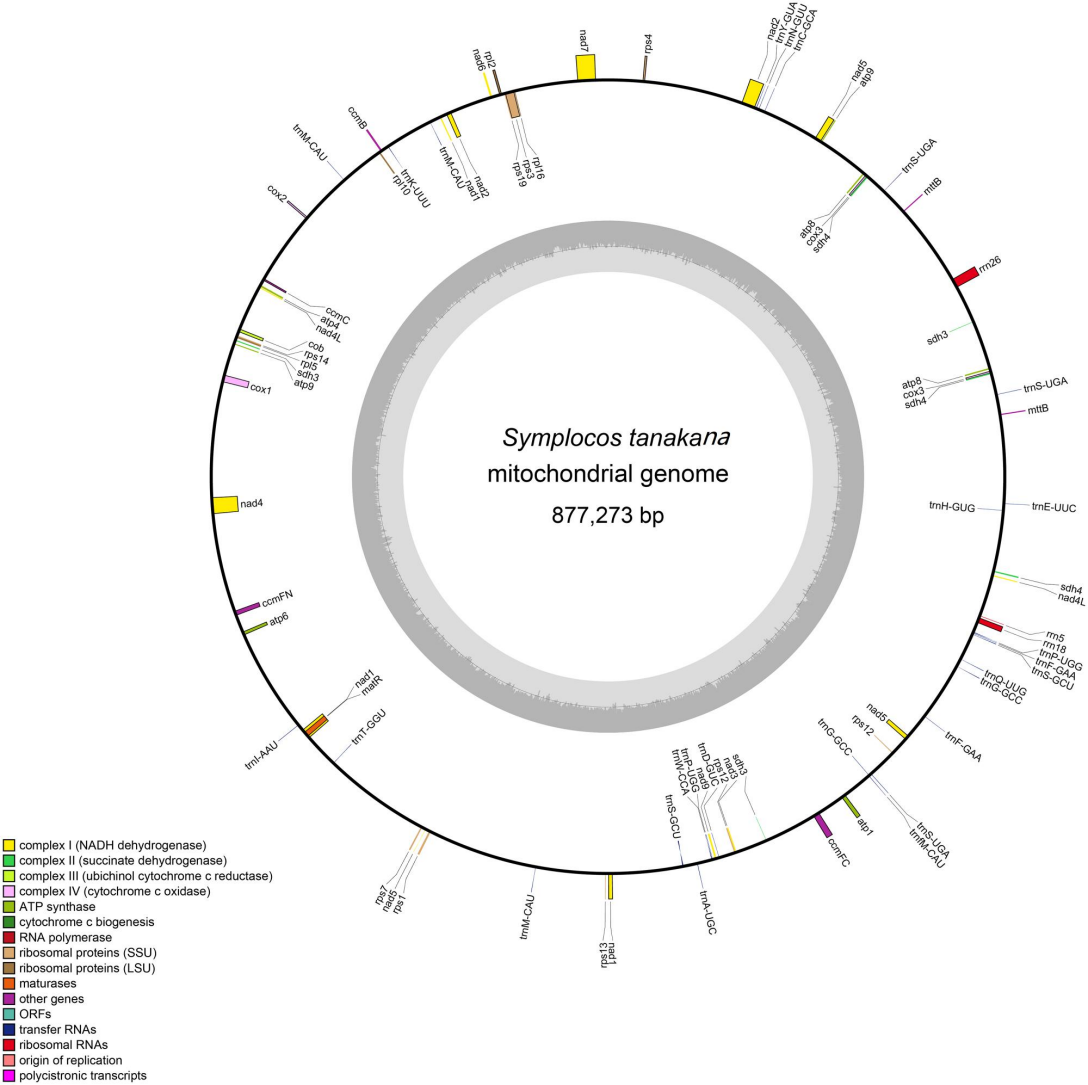
Map of the *symplocos tanakana* mitochondrial genome representing genes and repeats. Genes shown in the outer circle are transcribed clockwise, and genes shown in the inner circle are transcribed counterclockwise. Repeats >500 bp and similarity >95% are represented.

The mitochondrial genome of *S. tanakana* was sequenced through both short-read and long-read sequencing. This resulted in 65.3 million reads and 9.79 GB of raw data. The assembled mitochondrial genome of *S. tanakana* forms a single circle. Its total length is 877,273 bp with 45.44% GC content. In total, mean sequencing depth was 209.52 × (Supplemental Figure 1). *S. tanakana* mitogenome annotation showed 68 genes comprising 38 protein-coding genes [ATP synthase (five genes), cytochrome C biogenesis (one genes), ubiquinol cytochrome reductase (four gene), cytochrome C oxidase (three genes), maturase (one gene), transport membrane protein (one gene), NADH dehydrogenase (nine genes), ribosomal proteins (LSU; four genes), ribosomal proteins (SSU; eight genes), and succinate dehydrogenase (two genes)] and 30 non-coding genes, which include 27 tRNA and 3 rRNA genes (Figure 2). Among these, eight protein-coding genes contain introns: *nad1*, *nad2*, *nad5*, and *nad7* harbor four introns each; *nad4* harbors two introns; and *rps3*, *ccmFC*, and *cox1* harbor one intron each. In total, the *S. tanakana* mitochondrial genome houses multiple copies of the following seven genes: *nad4L*, *cox3*, *atp1*, *atp8*, *atp9*, *sdh4*, *and mttB*. Additionally, there are complex gene structures, including cis-splicing (*nad7*, *rps3*, *cox1*, *nad4*, and *ccmFC*) (Supplemental Figure 2) and trans-splicing genes (*nad5*, *nad1*, and *nad2*) (Supplemental Figure 3). The total lengths of the protein-coding and non-coding genes are 35,511 bp and 7437 bp, respectively, accounting for 4.05% and 0.84% of the total genome length.

### Phylogenetic analysis

**Figure 3.**
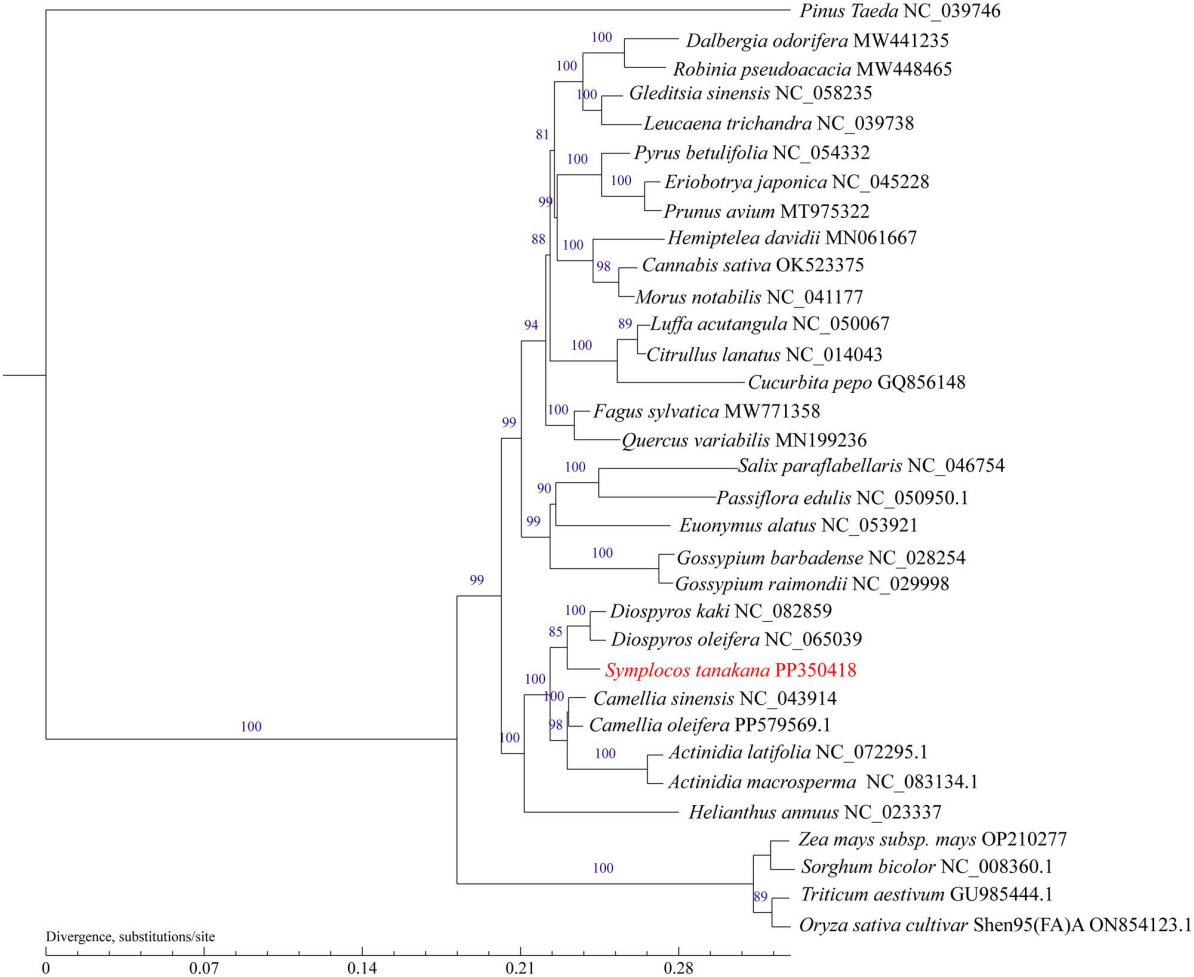
Data matrix constructed using a maximum likelihood tree based on 13 shared genes. These genes were *atp1*, *atp4*, *atp9*, *nad2*, *nad3*, *nad4*, *nad4L*, *ccmB*, *ccmC*, *ccmFC*, *ccmFN, cob*, and *matR* of *symplocos tanakana* and 32 other species. The bootstrap values are indicated on each node. The mitogenome sequence of *S. tanakana* in this study is highlighted in red. The following sequences were used: NC_039746, OK523375, NC_041177, MN061667, NC_054332, NC_045228, MT975322, NC_014043 (Alverson et al. 2010), NC_050067 (Ruang-areerate et al. 2020), GQ856148, NC_058235, NC_039738, MW448465, MW441235, NC_028254, NC_029998, NC_053921, NC_046754, NC_043914, NC_065039 (Xu, 2022), NC_082859, NC_023337 (Bock et al. 2014), MW771358 (Mishra et al. 2021), NC_083134.1, PP579569.1 (Xiao, 2024), NC_072295.1, NC_050950.1 (Yang and Wang, 2020), GU985444.1, NC_008360.1, ON854123.1, OP210277, and MN199236.

Phylogenetic analysis was conducted using the maximum likelihood (ML) method, based on the mitochondrial genome sequences of *S. tanakana* and those of 32 other species, whose mitochondrial genomes are deposited in GenBank, to elucidate the genetic status of *S. tanakana.* Among these species, *Pinus taeda* (NC_039746) from the Pinaceae family was utilized as an outgroup, as illustrated in Figure 3. The results indicate that *S. tanakana* is most closely related to *Diospyros kaki* (NC_082859) and *Diospyros oleifera* (NC_065039), all of which belong to the family of Ericales.

## Discussion and conclusion

*S. tanakana* is an economically and ecologically important plant. However, to the best of our knowledge, the mitochondrial genome had not been reported for any of the Symplocaceae species. Hence, we have assembled and annotated the mitogenome of *S. tanakana* as the first representative of this family. The mitochondrial genome consisted of a single circular chromosomal structure comprising 877,273 bp. In total, 290 ORFs and 68 genes were annotated, including 38 protein-coding, 3 rRNA, and 27 tRNA genes. According to published plant mitogenome sequence data, plant mitochondrial genomes display high variability in shape and structure and exist in single circular, multiple circular, or non-circular forms because of homologous recombination between repeats (Handa 2008; Bock and Knoop 2012; Gualberto et al. 2014; Liu et al. 2022). For example, although the mitochondrial genome of the hybrid A1 lines of *Saccharum* spp. (Zhou et al. 2022), *Juglans mandshurica* (Su et al. 2023), *Castanea mollissima* Blume (Guo et al. 2023), and *Allium fistulosum* (Tsujimura et al. 2019) exist as two distinct circular chromosomes, those of *Populus deltoides* (Qu et al. 2023) and *Allium cepa* (Hui et al. 2020*)* exist as three circular chromosomes. Plant mitogenomes are being increasingly used in phylogenetic studies owing to their evolutionary relevance with regard to the genetic information that they carry. In the present study, we constructed a phylogenetic tree using PhyML v3.0 based on 13 shared mitochondrial genes from 33 species. The ML tree, derived from the mitochondrial genome of *S. tanakan*a, effectively distinguished and classified dicotyledons and monocotyledons, particularly highlighting the separation of angiosperms and gymnosperms. Phylogenetic clustering and genetic distance calculations indicated that *S. tanakana* exhibits the closest evolutionary affinity with *Diospyros kaki* (NC_082859) and *Diospyros oleifera* (NC_065039). Both of these species belong to the Ebenaceae family and are clustered together in the phylogenetic tree. Notably, the phylogenetic tree based on mitochondrial DNA is consistent with the latest classification by the Angiosperm Phylogeny Group (APG). Thus, the complete mitochondrial genomic sequence of *S. tanakana* presented in this study offers valuable insights for further evolutionary investigations within the Symplocaceae family and the development of molecular markers for *S. tanakana.*

## Supplementary Material

Figure 3 trans gene Supplementary material.tif

Figure 2 cis splicing gene Supplementary material.tif

Result of DNA gel electrophoresis Supplementary material M mark 1sample.tiff

Figure1Sequencing Depth and Coverage Map Supplementary material.tif

## Data Availability

The genome sequence data are openly available in GenBank of NCBI at (https://dataview.ncbi.nlm.nih.gov/) under the accession no. PP350418. The associated BioProject, SRA, and Bio-Sample numbers are PRJNA1083057, SRR28204468, and SAMN40231565, respectively.
