# Supplementary figures and images for: The complete mitochondrial genome of Symplocos tanakana
Source: Mitochondrial DNA B Resour. 2025 Jul 10;10(8):692–7. doi: 10.1080/23802359.2025.2528579 (PMC12247094; doi:10.1080/23802359.2025.2528579)

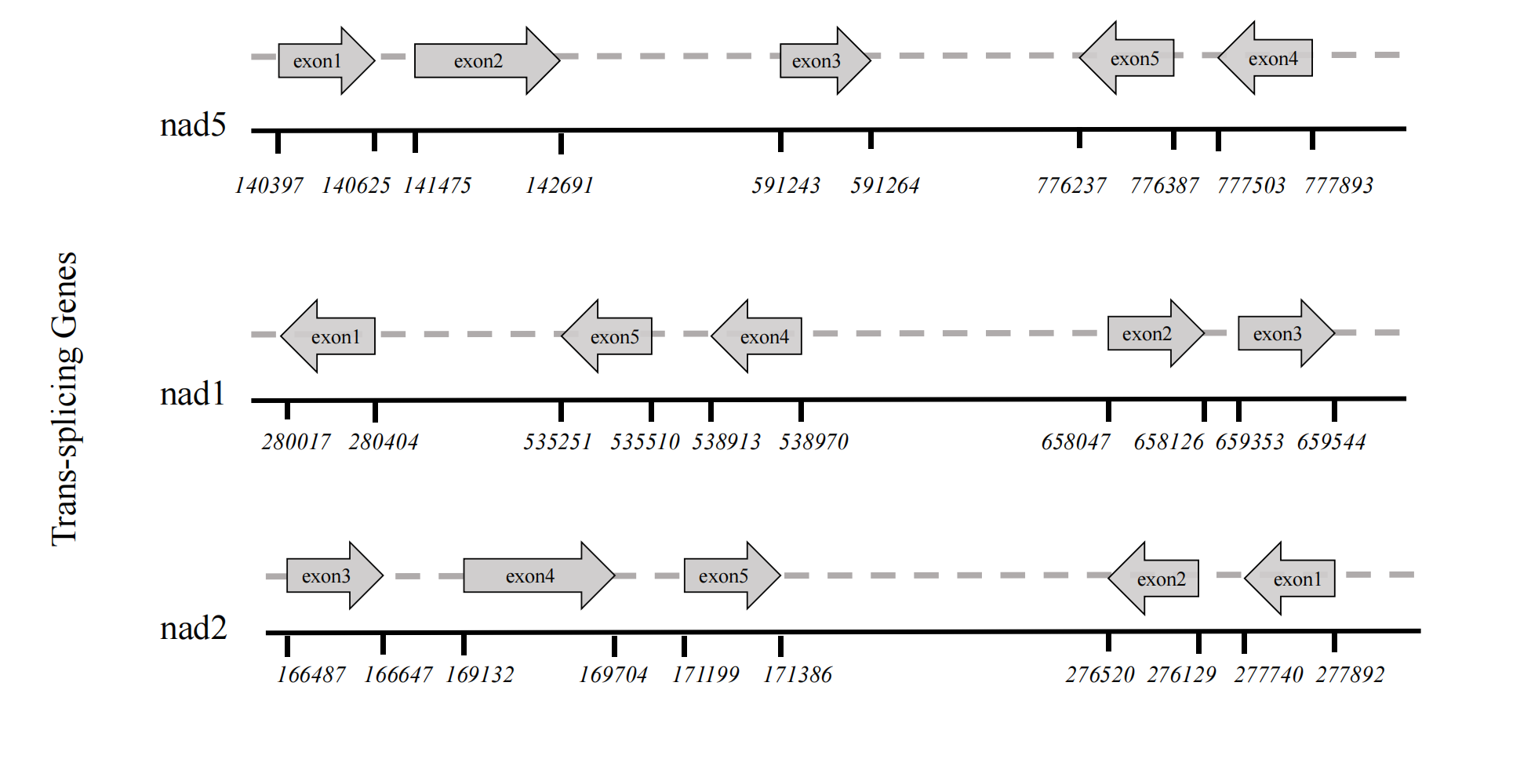

Supplement: Figure 3 trans gene Supplementary material.tif [file TMDN_A_2528579_SM8591.tif]

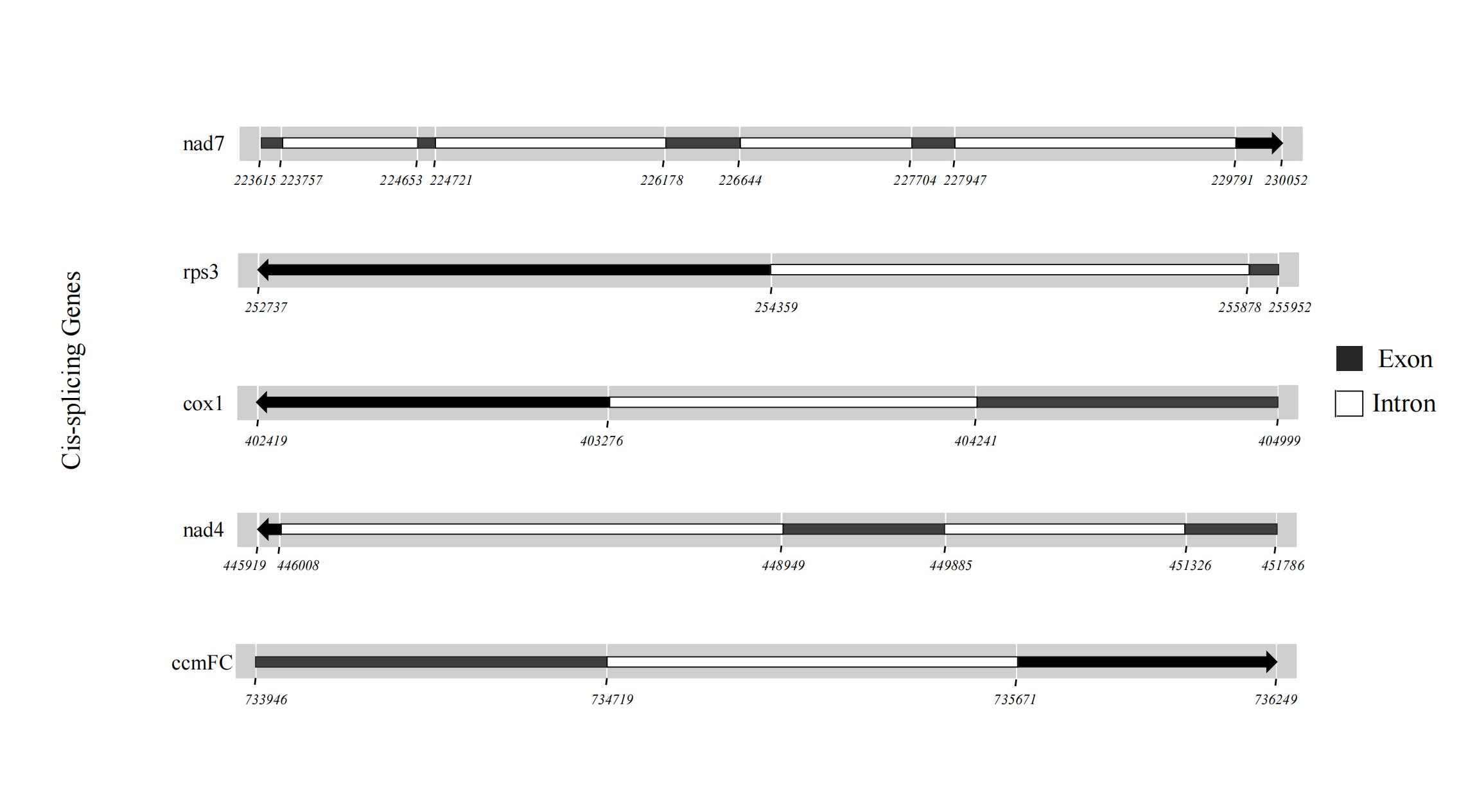

Supplement: Figure 2 cis splicing gene Supplementary material.tif [file TMDN_A_2528579_SM8590.tif]

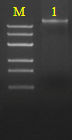

Supplement: Result of DNA gel electrophoresis Supplementary material M mark 1sample.tiff [file TMDN_A_2528579_SM8589.tiff]

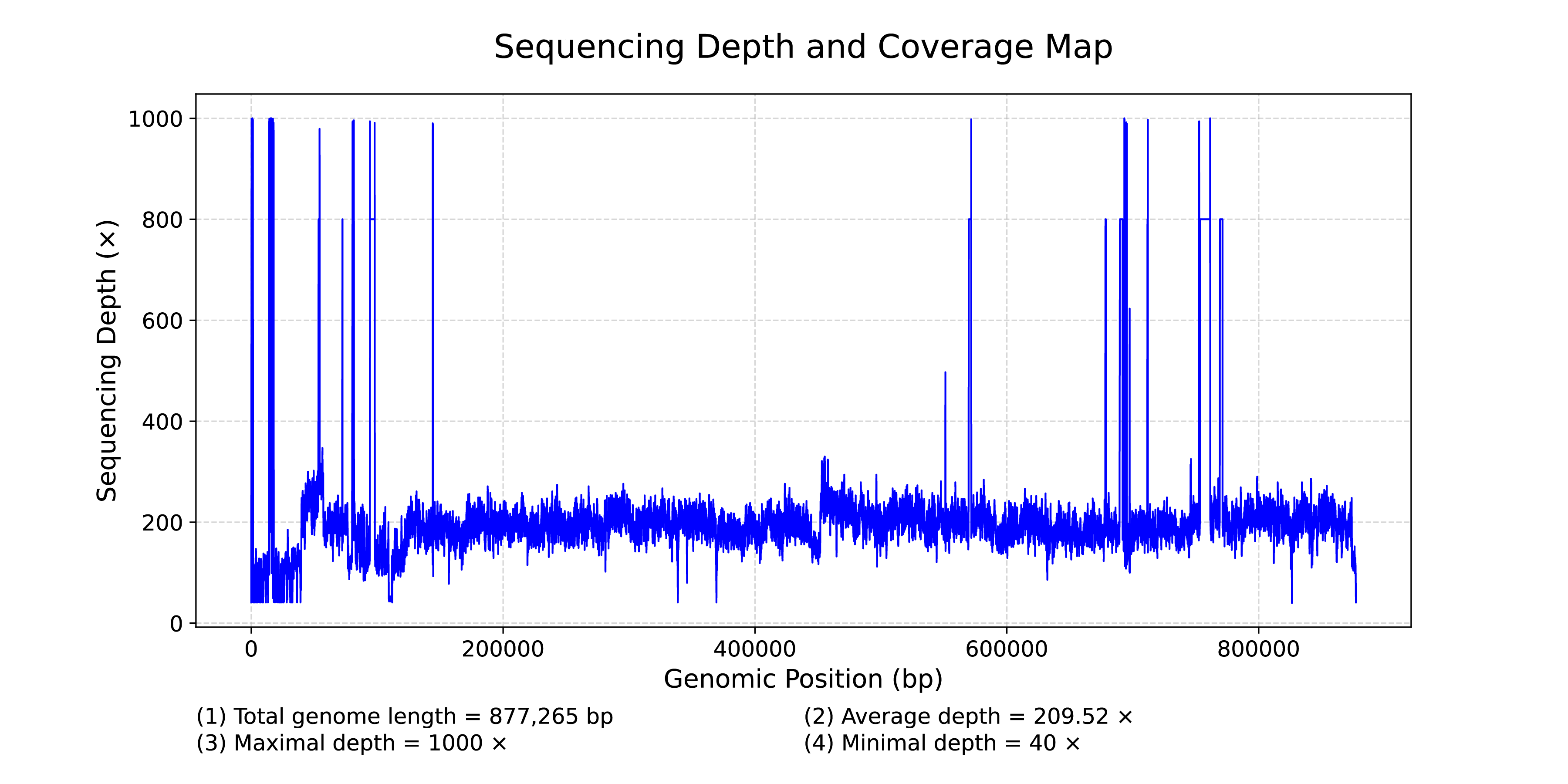

Supplement: Figure1Sequencing Depth and Coverage Map Supplementary material.tif [file TMDN_A_2528579_SM8588.tif]
